# The Association Between Weight‐Adjusted‐Waist Index and S‐Klotho Levels in Adults: NHANES 2007–2016

**DOI:** 10.1002/fsn3.70487

**Published:** 2025-07-17

**Authors:** Jingjing Chen, Qingyi Zeng, Li Liu, Yilan Li, Anning Wang, Yingqi Yi, Zhanglan Wang, Weihong Sun, Wei Zhou, Yun Ye, Wei Li

**Affiliations:** ^1^ Department of Cardiovascular Medicine Affiliated Hospital of Guizhou Medical University Guiyang China; ^2^ Department of Cardiovascular Medicine Guizhou Provincial People's Hospital Guiyang China

**Keywords:** National Health and Nutrition Examination Survey (NHANES), Serum Klotho levels, weight‐adjusted waist index (WWI)

## Abstract

The weight‐adjusted waist index (WWI) is a new obesity metric that may better reflect body fat distribution than traditional measures like body mass index (BMI). However, the link between WWI and serum Klotho levels, an aging biomarker, is not well understood. The study involved non‐pregnant adults aged 40 to 79 years who had comprehensive data regarding BMI, waist circumference (WC), and serum Klotho levels. We analyzed data from 12,809 participants aged 40 to 79 years from NHANES. WWI was calculated by dividing WC by the square root of body weight. Participants were categorized into quartiles based on WWI values. A multivariate linear regression model assessed the relationship between WWI and serum Klotho levels, with subgroup analyses for demographic consistency. The analysis showed a significant negative correlation between WWI and serum Klotho levels (*β* = −24.75, 95% CI: −24.84, −24.66). Participants in the highest WWI quartile had lower serum Klotho levels than those in the lowest quartile (*β* = −27.88, 95% CI: −28.03, −27.73). This negative association was consistent across all subgroups, including age, gender, diabetes status, hypertension status, and BMI categories. Our findings indicate a significant negative association between WWI and serum Klotho concentrations, suggesting that WWI may be a practical clinical indicator for identifying individuals at higher risk of accelerated aging and related metabolic complications. Incorporating WWI into routine assessments could help clinicians better associated with patients at risk for reduced Klotho and its associated health consequences, supporting more targeted prevention or intervention strategies. The cross‐sectional design precludes causal inference, and potential confounders such as physical activity, dietary factors, and medication use were not fully accounted for. Further longitudinal and mechanistic studies are needed to confirm these associations and clarify underlying pathways.

## Graphical abstract

This study investigates the relationship between the weight‐adjusted waist index (WWI) and serum Klotho levels in adolescents, highlighting WWI as a potentially more accurate obesity indicator than traditional measures like BMI. The findings reveal a significant negative association between WWI and serum Klotho concentrations, suggesting that WWI may serve as a predictive indicator of serum Klotho levels, with implications for understanding obesity's influence on aging. Further research is needed to explore the underlying mechanisms of this relationship.

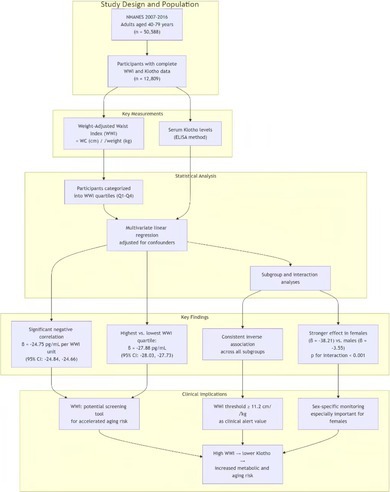

## 1 Introduction

Serum Klotho has emerged as a significant biomarker for aging, offering valuable insights into the biological processes that underpin the aging phenomenon (Abraham and Li 2022). As the global population continues to age, accurately detecting and monitoring aging becomes increasingly crucial. Understanding and utilizing markers like Klotho can play a pivotal role in developing strategies to delay the aging process and improve overall healthspan.

Obesity, which is characterized by an accumulation of excess body fat, cannot be evaluated exclusively using body mass index (BMI) because it yields inconsistent results. BMI fails to account for fat distribution, leading to different fat amounts in individuals with the same BMI (Shungin et al. 2015). Research indicates that trunk fat may be more harmful than fat in other areas (Rask‐Andersen et al. 2019).

Traditional obesity indices such as BMI and waist circumference (WC) have significant limitations. BMI fails to differentiate between fat and lean mass, and WC, though reflective of central fat, is affected by overall body size. In contrast, weight‐adjusted waist index (WWI) divides WC by the square root of body weight, thus minimizing the confounding effect of body mass and better representing central adiposity and fat redistribution, especially in older adults. Recent studies have shown WWI to be a superior predictor of cardiometabolic risk and mortality compared to BMI and WC (Park et al. 2018; Wen et al. 2025).

Emerging evidence indicates that lower serum Klotho levels are associated with increased carotid intima‐media thickness (CIMT) and epicardial fat thickness (EFT), as well as reduced brachial artery flow‐mediated dilation (FMD), suggesting its potential as a predictive biomarker for atherosclerosis development (Keles et al. 2015).

Notably, recent epidemiological investigations reveal a U‐shaped association between WWI and bowel dysfunction: higher WWI levels have been linked to an increased risk of chronic diarrhea, while WWI values below 9.77 correlate with an elevated risk of chronic constipation (Yang and Sun 2024). These findings position WWI as a robust indicator for assessing intestinal health status in the United States adult population.

Nonetheless, studies exploring the connection between the WWI and serum Klotho, which serves as a biomarker for aging, are limited. The specific relationship between WWI and serum Klotho levels remains underexplored in current research. Our study bridges this knowledge gap by systematically investigating the WWI‐Klotho association through multivariable‐adjusted analyses of nationally representative data.

This paper intends to examine the association between WWI and serum Klotho concentrations in adults, offering a more straightforward approach to tracking aging and deepening the comprehension of how obesity influences the aging process, thereby promoting better health outcomes.

## 2 Methods

### 2.1 Data Source and Study Population

The National Health and Nutrition Examination Survey (NHANES) represents a detailed cross‐sectional study conducted in the United States by the National Center for Health Statistics (NCHS) under the auspices of the CDC. This investigation employed data gathered from five distinct survey cycles (2007–2008, 2009–2010, 2011–2012, 2013–2014, and 2015–2016) to assess the WWI and serum Klotho concentrations. Approval for the NHANES protocol was granted by the NHRS Research Ethics Review Board, and the methods for data collection are accessible to the public (https://www.cdc.gov/nchs/nhanes/index.htm).

The study involved non‐pregnant adults between the ages of 40 and 79 who had comprehensive data regarding BMI, body weight, WC, 25‐hydroxyvitamin D (25(OH)D), and cardiovascular disease (CVD). From a total of 50,588 participants, 12,809 adults with available serum Klotho and WWI information were included in the analysis.

### 2.2 Measurement of WWI and Serum Klotho Levels

The WWI is determined by dividing the WC measured in centimeters by the square root of body weight in kilograms (cm/√kg). The BMI is calculated based on height and weight data collected at Mobile Examination Centers (MECs). Participants were classified into three categories: normal weight (BMI ≥ 18.5 and < 25 kg/m^2^), overweight (BMI ≥ 25 and < 30 kg/m^2^), and obese (BMI ≥ 30 kg/m^2^).

Serum Klotho concentrations were assessed using frozen samples that were collected from 2007 to 2016, with the analyses performed in 2019 and 2020. These specimens were maintained at a temperature of −80°C. The CDC forwarded the samples to the University of Washington, where testing was carried out using an enzyme‐linked immunosorbent assay (ELISA) kit provided by IBL International. To ensure quality control, the mean of two duplicate tests was calculated, and any samples showing a variance greater than 10% were subjected to reanalysis.

### 2.3 Covariates

Potential confounding variables were selected based on prior research and their biological significance. The covariates analyzed in this study included age, gender, race/ethnicity, BMI, smoking habits, alcohol intake, hypertension, diabetes, chronic kidney disease (CKD), and CVD. Additionally, we assessed levels of 25(OH)D, total cholesterol (TC), triglycerides (TG), creatinine, serum calcium, and phosphorus. Race/ethnicity was classified into categories such as Mexican American, other Hispanic, Non‐Hispanic White, Non‐Hispanic Black, and others. Alcohol consumption was defined as having consumed at least 12 alcoholic beverages over the past year. Participants were identified as current smokers if they responded “yes” to the question, “Do you currently smoke cigarettes?” Education levels were categorized based on college attendance, while marital status was classified as either married or cohabiting versus single. Income was categorized according to whether the annual household income was above or below $20,000. A history of cardiovascular disease was determined by positive responses to questions regarding congestive heart failure, coronary heart disease, heart attack, or stroke, with CVD defined as the presence of any of these conditions.

The covariates for this analysis were selected based on previous literature that indicates their influence on both WWI and serum Klotho levels. These include age, gender, race/ethnicity, BMI, smoking habits, and chronic conditions such as diabetes and hypertension.

### 2.4 Statistical Analyses

Three sequential models were constructed: (1) Crude model without adjustment; (2) Demographically adjusted model (age, sex, race/ethnicity); (3) Fully adjusted model incorporating cardiovascular risk factors (hypertension, diabetes), renal function (CKD status), and mineral metabolism markers (calcium, phosphorus). Outliers, defined as values more than 3 standard deviations from the mean for key continuous variables, were examined.

Sensitivity analyses included: (1) Variance inflation factors (VIFs) to quantify multicollinearity; (2) Complete‐case analysis excluding missing data; (3) Stratified models by CKD status (eGFR < 60 vs. ≥ 60 mL/min/1.73 m^2^).

To ensure robustness and reliability, we conducted sensitivity analyses to address potential overfitting. We compared models of varying complexity by minimizing the Akaike information criterion (AIC) and the Bayesian information criterion (BIC), thus ensuring model selection was not only appropriate but also justified. This approach allowed us to determine which covariates contributed meaningfully to the predictive power of our models without introducing unnecessary complexity. Data integrity checks were performed and these outliers were excluded in sensitivity analyses. No material changes to the main findings were observed after their exclusion.

We formally tested effect modification by including multiplicative interaction terms between WWI and each subgroup variable (gender, age group, diabetes status, hypertension status, BMI category) in multivariable linear regression models. The statistical significance of interactions was assessed using Wald tests with Bonferroni correction for multiple comparisons (*α* = 0.01).

Statistical evaluations were performed utilizing IBM SPSS Statistics software (Version 22, IBM Corp., USA). Continuous variables were expressed as means with standard deviations (SD), whereas categorical variables were shown as frequencies and percentages. The relationship between the WWI and serum Klotho levels was analyzed using a weighted linear regression model, and further subgroup and interaction analyses were conducted for a more comprehensive examination.

## 3 Results

### 3.1 Baseline Characteristics of Study Participants

A total of 12,809 individuals aged 40 to 79 years participated in this study. The average serum Klotho levels across the WWI quartiles (Q1–Q4) were 885.98, 857.55, 843.31, and 835.41 pg/mL, indicating significant differences (*p* < 0.001). The quartiles also exhibited notable variations in age, race/ethnicity, gender, BMI, hypertension, diabetes, CKD, CVD, TC, smoking habits, alcohol intake, TG, phosphorus, 25(OH)D, education level, marital status, and income. Detailed baseline characteristics by WWI quartiles are presented in Table 1.

**TABLE 1:** Characteristics of individuals by WWI quartile from NHANES (2007–2016).

Characteristics	Weight‐adjusted waist index				
	Q1	Q2	Q3	Q4	*p*
Klotho	885.98 ± 326.64	857.55 ± 302.05	843.31 ± 299.93	835.41 ± 306.71	< 0.001
Age, years	52.82 ± 9.70	56.20 ± 10.33	59.19 ± 10.44	62.17 ± 10.41	< 0.001
Sex, male, *n* (%)	1833 (29.5)	1770 (28.5)	1538 (24.7)	1075 (17.3)	< 0.001
Race/ethinicity					< 0.001
Mexican American	266 (13.0)	472 (23.0)	609 (29.7)	706 (34.4)	
Other Hispanic	274 (18.3)	373 (24.9)	413 (27.6)	435 (29.1)	
Non‐Hispanic White	1417 (25.7)	1379 (25.0)	1324 (24.0)	1400 (25.4)	
Non‐Hispanic Black	862 (34.3)	642 (25.6)	540 (21.5)	466 (18.6)	
Others	364 (29.6)	365 (29.7)	279 (22.7)	223 (18.1)	
BMI (kg/m^2^)	26.21 ± 4.78	28.66 ± 5.28	30.52 ± 5.78	33.74 ± 7.28	< 0.001
Hypertension, *n* (%)	980 (16.6)	1357 (23.0)	1586 (26.9)	1977 (33.5)	< 0.001
Diabetes, *n* (%)	224 (9.9)	401 (17.7)	629 (27.8)	1007 (44.5)	< 0.001
CKD, *n* (%)	61 (13.1)	90 (19.4)	128 (27.6)	185 (39.9)	< 0.001
CVD, *n* (%)	180 (11.7)	331 (21.4)	412 (26.7)	621 (40.2)	< 0.001
TC (mmol/L)	5.15 ± 1.01	5.24 ± 1.08	5.16 ± 1.15	5.07 ± 1.15	< 0.001
Current smoking, *n* (%)	574 (28.0)	514 (25.1)	472 (23.0)	490 (23.9)	< 0.001
Alcohol consumption, *n* (%)	2307 (27.0)	2253 (26.4)	2112 (24.7)	1868 (21.9)	< 0.001
Creatinine (μmol/L)	82.13 ± 38.88	81.13 ± 39.12	81.01 ± 44.65	78.91 ± 39.31	0.013
TG (mmol/L)	1.57 ± 1.50	1.88 ± 1.50	2.04 ± 1.86	2.12 ± 1.54	< 0.001
Calcium (mmol/L)	2.35 ± 0.09	2.35 ± 0.09	2.35 ± 0.09	2.35 ± 0.10	0.789
Phosphorus (mmol/L)	1.20 ± 0.18	1.20 ± 0.18	1.20 ± 0.18	1.21 ± 0.18	0.001
25(OH)D (nmol/L)	68.80 ± 26.42	66.81 ± 26.42	65.79 ± 26.64	64.56 ± 28.69	< 0.001
CVD	176 (11.4)	331 (21.4)	416 (26.9)	621 (40.2)	< 0.001
Ever attended college, *n* (%)	1933 (30.1)	1729 (27.0)	1496 (23.3)	1257 (19.6)	< 0.001
Married/living with a partner, *n* (%)	2162 (25.9)	2218 (26.6)	2110 (25.3)	1844 (22.1)	< 0.001
Annual income > $20,000, *n* (%)	2596 (27.0)	2539 (26.4)	2333 (24.3)	2150 (22.4)	< 0.001

*Note:* Values are presented as mean ± SD or number (percentage).

Abbreviations: BMI, body mass index; CKD, chronic kidney disease; CVD, cardiovascular disease; TC, total cholesterol; TG, triglyceride.

### 3.2 Association Between WWI and Klotho

We constructed three linear regression models to evaluate the impact of WWI on serum Klotho levels (Table 2). All models revealed an inverse relationship between WWI and serum Klotho concentration. After adjusting for age, sex, race/ethnicity, CVD, vitamin D, CKD, hypertension, diabetes, calcium, and phosphorus, a one‐unit increase in WWI was associated with a decrease of 24.75 pg/mL in serum Klotho (Model 3: *β* = −24.75, 95% CI: −24.84, −24.66). This association remained significant when WWI was analyzed in quartiles; specifically, a one‐unit increase in WWI corresponded to a 27.88 pg/mL reduction in serum Klotho for participants in quartile 4 compared to those in quartile 1 (*β* = −27.88, 95% CI: −28.03, −27.73; *p* for trend < 0.001).

**TABLE 2:** Association between weight‐adjusted waist index and S‐Klotho.

Exposure	Model 1 (*β* [95% CI])	*p*	Model 2 (*β* [95% CI])	*p*	Model 3 (*β* [95% CI])	*p*
WWI (continuous)	−26.90 (−26.98, −26.83)	< 0.001	−22.24 (−22.32, −22.15)	< 0.001	−24.75 (−24.84, −24.66)	< 0.001
WWI						
Quartile 1	Reference		Reference		Reference	
Quartile 2	−2.05 (−2.18, −1.92)	< 0.001	−2.13 (−2.25, −2.00)	< 0.001	−2.46 (−2.59, −2.34)	< 0.001
Quartile 3	−9.62 (−9.75, −9.49)	< 0.001	−4.35 (−4.48, −4.22)	< 0.001	−5.04 (−5.18, −4.91)	< 0.001
Quartile 4	−33.05 (−33.19, −32.91)	< 0.001	−26.41 (−26.55, −26.27)	< 0.001	−27.88 (−28.03, −27.73)	< 0.001
*p* for trend	< 0.001		< 0.001		< 0.001	

*Note:* Model 1: no covariates were adjusted. Model 2: age, sex, and race/ethnicity were adjusted. Model 3: age, sex, and race/ethnicity, CVD, vitamin D, CKD, hypertension, diabetes mellitus, calcium, phosphorus.

Abbreviations: CKD, chronic kidney disease; CVD, cardiovascular disease.

### 3.3 Model Selection

**FIGURE 1:**
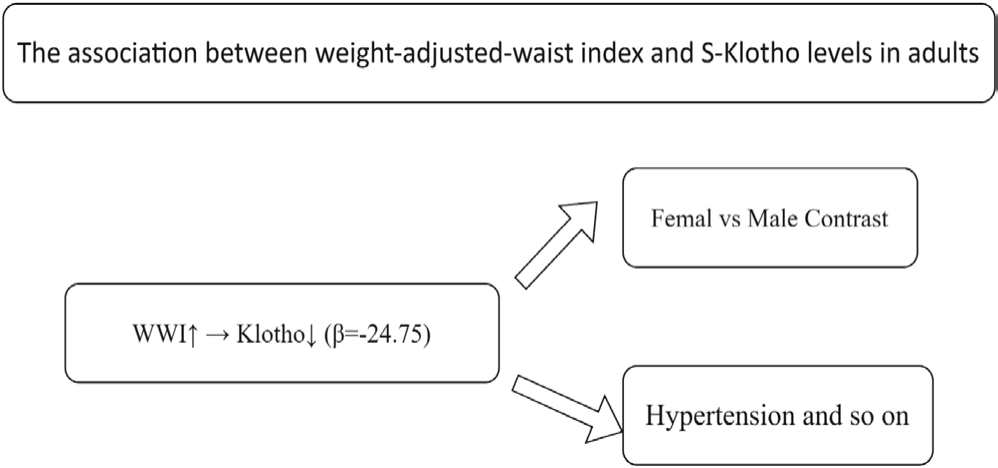
Flow chart of study patients. NHANES, National Health and Nutrition Examination Survey; WWI, weight‐adjusted‐waist index.

In this study, we performed model selection using the AIC and BIC to assess our multivariate linear regression models for predicting Klotho levels (Figure 1). The models compared were as follows:

Model 1 included basic covariates: weight, WC, age, and gender. This model yielded an AIC of 188,340.28 and a BIC of 188,377.71.

Model 2 included additional covariates, such as alcohol consumption and education level, improving the model significantly with an AIC of 104,111.94 and a BIC of 104,160.25.

The results indicated that Model 2 provided a better fit based on both AIC and BIC values, suggesting that the inclusion of additional covariates enhances the predictive capability regarding Klotho levels. We selected covariates based on existing literature, emphasizing their relevance to Klotho levels and considering potential confounders.

### 3.4 Subgroup Analysis

We chose these specific covariates because they are known to influence both WWI and serum Klotho levels. For example, BMI is a commonly utilized obesity metric that correlates with body composition, while chronic conditions like diabetes and hypertension can impact metabolic pathways associated with Klotho expression. The selection of these covariates aimed to adjust for variables that might confound the relationship between WWI and Klotho levels.

Subgroup analyses were conducted based on gender, age, diabetes mellitus, hypertension, and BMI (Table 3). Consistent findings were observed across these subgroups in the adjusted models. For women, a one‐unit increase in WWI was linked to a decrease of 38.21 pg/mL in serum Klotho (*β* = −38.21, 95% CI: −38.32, −38.09). In men, a one‐unit increase in WWI was associated with a decrease of 3.55 pg/mL in serum Klotho (*β* = −3.55, 95% CI: −3.68, −3.42). Furthermore, a significant inverse relationship between WWI and serum Klotho was found when stratified by age, diabetes status, BMI, and hypertension. Across all BMI categories, a higher WWI was linked to lower serum Klotho concentrations.

**TABLE 3:** Subgroup analysis for the association between weight‐adjusted waist index and S‐Klotho.

	*β* (95% CI)	*p*
Stratified by gender		
Male	−3.55 (−33.68, −3.42)	< 0.001
Female	−38.21 (−38.32, −38.09)	< 0.001
Stratified by age		
< 60	−24.53 (24.63, −24.42)	< 0.001
≥ 60	−28.53 (−28.66, −28.40)	< 0.001
Stratified by diabetes mellitus		
No	−26.44 (−26.53, −26.34)	< 0.001
Yes	−28.41 (−28.64, −28.17)	< 0.001
Stratified by BWI		
18.5 ≤ BWI < 25	−39. 15 (−39.36, −38.95)	< 0.001
25 ≤ BWI < 30	−19.42 (−19.61, −19.24)	< 0.001
BWI ≥ 30	−13.33 (−13.46, −13.20)	< 0.001
Stratified by hypertension		
No	−17.10 (−17.21, −16.99)	< 0.001
Yes	−33.72 (−33.86, −33.59)	< 0.001

*Note:* Age, sex, and race/ethnicity, CVD, vitamin D, CKD, hypertension, diabetes mellitus, calcium and phosphorus were all adjusted except the variable itself.

Abbreviations: BMI, body mass index; CKD, chronic kidney disease; CVD, cardiovascular disease.

A significant sex‐specific modification was observed in the WWI‐Klotho association (*p* < 0.001), with females demonstrating stronger inverse relationships (*β* = −28.91, 95% CI: −43.5 to −14.3) compared to males. Hypertension status also modified this association (*p* = 0.012), as detailed in Table 4.

**TABLE 4:** Subgroup analysis of WWI‐Klotho associations with interaction terms.

Interaction term	*β* (95% CI)	SE	*p*
Female	−28.91 (−43.5, −14.3)	7.43	< 0.001
Age ≥ 60	−5.17 (−10.8, 0.46)	2.86	0.072
Diabetes	1.05 (−2.1, 4.2)	1.59	0.510
Hypertension	−6.88 (−12.3, −1.5)	2.73	0.012

## 4 Discussion

Our cross‐sectional study identified a significant negative correlation between WWI and serum Klotho levels in adults aged 40 to 79 years. This association was consistent across various subgroups, including age, gender, diabetes status, hypertension, and BMI, with a more pronounced effect observed in females. The underlying mechanism for this difference remains unclear, but it could potentially involve sex hormones or variations in body composition. To ensure robustness and reliability, we conducted sensitivity analyses to address potential overfitting.

The widely accepted criteria for defining obesity typically encompass BMI and WC. Francisco and his team reported an association between body composition and serum Klotho concentrations (Amaro‐Gahete et al. 2019). They found that the lean mass index was positively associated with serum Klotho levels, as indicated by data from 74 middle‐aged sedentary individuals. The WWI, a distinctive obesity metric identified by Park et al. in their 2018 study involving 456,629 South Koreans, was linearly associated with both cardiometabolic morbidity and mortality, in contrast to BMI. Additionally, WWI demonstrated stronger predictive value for CVD mortality. In clinical practice, BMI and WC are widely utilized indicators of obesity. Recent research suggests that alterations in body composition, such as reductions in lean mass and elevations in fat mass, are associated with bone mineral density (Shieh et al. 2023). In the current study, we observed that higher WWI was associated with lower serum Klotho levels across all BMI categories. This finding aligns with earlier research that emphasized the shortcomings of BMI in assessing individual health risks (Kalkhoff et al. 1983; Kissebah et al. 1982).

Emerging evidence suggests that serum Klotho may act as a prominent anti‐obesogenic regulator (Landry et al. 2021). In individuals with obesity and diabetes, lower serum Klotho levels were associated with compromised whole‐body energy metabolism (Amitani et al. 2013; Tang et al. 2023). Moreover, the administration of Klotho led to a reduction in lipid buildup in both the liver and adipose tissue, an increase in lean mass, and a rise in energy expenditure, all without altering food intake or body weight in obese mice (Rao et al. 2019). In a study of 107 girls (mean age 8.4 ± 1.8 years) who were experiencing weight gain, researchers found inverse associations between serum Klotho levels and BMI, WC, body fat, and visceral fat both at baseline and after 5 years of follow‐up. For school‐aged girls, serum Klotho might serve as a protective factor against the buildup of visceral fat (Carreras‐Badosa et al. 2023). Reduced serum Klotho levels were associated with greater epicardial fat thickness, which is a form of visceral fat located around the heart, especially on the free wall of the right ventricle, the apex of the left ventricle, and the atrium (Keles et al. 2015). Epicardial fat thickness is associated with coronary artery disease and subclinical atherosclerosis. In this study, we explored the connection between serum Klotho levels and the WWI, a new and simple method for assessing cardiometabolic risk. Our findings revealed that individuals with lower Klotho levels exhibited significantly elevated WWI.

WWI offers a more precise evaluation of central obesity (Gavin and Bessesen 2020). It is well established that body fat distribution varies between males and females, a conclusion also supported by our research. We observed that the association between serum Klotho and WWI is more pronounced in females compared to males. Our gender‐stratified analysis revealed a stronger inverse WWI‐Klotho association in females. Women exhibit higher gluteofemoral subcutaneous fat deposition compared to males (Heid et al. 2010), which may buffer against visceral adiposity's inflammatory effects until WWI thresholds are exceeded. This aligns with our observed nonlinear dose–response pattern where the WWI‐Klotho association intensified above WWI ≥ 12.3 in females.

These findings are consistent with earlier genome‐wide association studies that identified sex‐specific differences in genetic loci associated with adiposity‐related anthropometric characteristics, including WC and waist‐to‐hip ratio (Heid et al. 2010; Randall et al. 2013; Winkler et al. 2015). Furthermore, genetic variations were noted exclusively for waist‐related phenotypes, with no differences found in height, weight, BMI, or hip circumference. The occurrence of higher BMI alongside a reduced risk of cardiometabolic diseases has been described through the concept of “favorable adiposity.” (Yaghootkar et al. 2016) Individuals carrying “favorable adiposity” alleles may have a greater ability of subcutaneous adipose tissue to accumulate excess calories in the form of lipids (Yaghootkar et al. 2014). This could help explain why individuals with elevated BMI and body fat percentage do not always show an increased risk of metabolic diseases. Among women with higher BMI but reduced cardiometabolic risk, there tends to be a greater accumulation of body fat in the lower body, as evidenced by a lower waist‐to‐hip ratio and larger hip circumference. In contrast, men are more likely to have excess body fat concentrated in the upper body, as indicated by a higher WC and waist‐to‐hip ratio (Yaghootkar et al. 2016). An elevated ratio of visceral to subcutaneous adipose tissue is linked to greater insulin resistance (Yaghootkar et al. 2014). WWI, as an indicator of visceral adiposity, demonstrated an inverse linear association with serum Klotho, suggesting a potential role of Klotho as a cardioprotective factor.

Emerging evidence suggests that Klotho acts as a multifunctional protein involved in regulating aging and metabolism. The findings elucidate that individuals with higher WWI exhibit lower levels of serum Klotho, suggesting a potential link between central obesity and metabolic dysfunction. This relationship highlights the need for further research to explore biological mechanisms underlying this association, as well as to evaluate the implications for obesity management in the context of aging.

Reduced serum Klotho levels are associated with a higher all‐cause mortality rate. While Klotho is mainly produced in the kidneys, it is also synthesized in the brain and various other tissues. Preclinical Klotho therapy has been shown to ameliorate cardiovascular disease as well as diabetes‐related conditions (Prud'homme et al. 2022). Previous studies have revealed that angiotensin II suppresses the expression of Klotho (Yoon et al. 2011), while losartan (an angiotensin II receptor antagonist) increased serum Klotho concentrations by 23% (Janić et al. 2019; Lim et al. 2014; Haussler et al. 2020). Vitamin D supplementation also elevates serum Klotho levels, potentially due to vitamin D binding to its receptor to upregulate Klotho transcription, as well as the complex relationship among the FGF23/Klotho/vitamin D axis (Edmonston et al. 2024).

The clinical significance of the observed differences among WWI quartiles is noteworthy. In our study, participants in the highest WWI quartile (Q4: 835.41 pg/mL) had substantially lower serum Klotho levels than those in the lowest quartile (Q1: 885.98 pg/mL), with a difference exceeding 50 pg/mL. Lower circulating Klotho has been associated with a higher risk of adverse health outcomes and all‐cause mortality (Keles et al. 2015; Yoon et al. 2011). Keles et al. (2015) found that individuals with atherosclerosis had significantly lower Klotho levels than controls, suggesting that even moderate changes in Klotho may translate to meaningful clinical risk.

In terms of practical implications, WWI reflects central adiposity more accurately than BMI (Park et al. 2018), and is linearly associated with cardiometabolic morbidity and mortality. Therefore, identifying individuals with high WWI may help clinicians recognize those at elevated risk of low Klotho and accelerated aging processes, who might benefit from more aggressive lifestyle interventions or monitoring.

A potential threshold for clinical WWI significance can be referenced from previous studies: Park et al. reported that a WWI of approximately 11.2 cm/√kg corresponded with increased metabolic risk in Asian populations (Park et al. 2018). In our study, Klotho levels declined most steeply above this threshold, especially in females (WWI ≥ 12.3). Thus, a WWI ≥ 11.2 cm/√kg could be viewed as an alert value in clinical practice for further metabolic or aging‐related risk assessment.

Our study demonstrated that the inverse association between WWI and serum Klotho levels is significantly stronger in females than in males, with a one‐unit increase in WWI leading to a much greater reduction in serum Klotho among women (*β* = −38.21 pg/mL) than men (*β* = −3.55 pg/mL).

Underlying this gender disparity may include differences in fat distribution, sex hormones, and genetic regulation. Women exhibit greater subcutaneous and gluteofemoral fat deposition, which may buffer against harmful metabolic effects until higher WWI thresholds are reached; this was supported by our observed nonlinear effect in females with WWI ≥ 12.3 (Keles et al. 2015; Gavin and Bessesen 2020). Sex hormones, particularly estrogen, positively regulate Klotho expression, and the decline of estrogen after menopause may accelerate central adiposity and Klotho reduction in women (Gavin and Bessesen 2020). Furthermore, genome‐wide association studies have revealed pronounced sex‐specific genetic effects on anthropometric traits (such as WC and waist‐hip ratio), further supporting the biological plausibility of gender‐specific associations (Gavin and Bessesen 2020; Heid et al. 2010; Randall et al. 2013; Winkler et al. 2015).

Clinical implications arise from the much steeper decline in Klotho level per WWI increment in women. This suggests WWI is a sensitive indicator for identifying women at higher risk of low Klotho and aging‐related comorbidities, especially above the clinical threshold of WWI (11.2–12.3 cm/√kg in our study and prior research, Park et al. 2018). Early identification using gender‐stratified thresholds could facilitate more proactive risk stratification and comprehensive intervention in females.

Several biological pathways may underlie the observed inverse association between the WWI and serum Klotho levels in adults: (1) Visceral Adiposity and Chronic Inflammation: WWI is a marker of central obesity, reflecting increased visceral fat. Visceral adipose tissue is metabolically active and secretes pro‐inflammatory cytokines, which can promote oxidative stress and accelerate aging processes. Chronic inflammation has been shown to downregulate Klotho expression and contribute to cardiovascular risk (Keles et al. 2015; Landry et al. 2021; Amitani et al. 2013; Rao et al. 2019). Individuals with higher WWI may thus experience greater systemic inflammation, resulting in suppressed Klotho synthesis and secretion (Keles et al. 2015; Landry et al. 2021; Rao et al. 2019). (2) Adipokines and Metabolic Dysfunction: Obesity alters adipokine profiles, reducing adiponectin and increasing leptin and resistin. These changes are linked to insulin resistance and metabolic syndrome (Landry et al. 2021). Klotho has been reported to counteract insulin resistance and improve lipid metabolism, and lower Klotho levels in obesity may represent a feedback response to persistent metabolic stress (Keles et al. 2015; Tang et al. 2023; Rao et al. 2019). (3) Direct Effects on Energy Metabolism: Experimental studies reveal that Klotho plays an anti‐obesogenic role by regulating whole‐body energy metabolism, increasing energy expenditure, and preserving lean mass (Landry et al. 2021; Rao et al. 2019). Animal models have shown that exogenous Klotho administration decreases lipid deposition in liver and adipose tissue, suggesting that reductions in Klotho might both reflect and contribute to worsening central fat accumulation (Rao et al. 2019). Longitudinal human data support an inverse relationship between Klotho and central adiposity, particularly visceral fat (Carreras‐Badosa et al. 2023). Role of Hormonal and Genetic Mediators: The FGF23‐Klotho axis, influenced by mineral metabolism (notably phosphate and vitamin D), may be perturbed in obesity (Edmonston et al. 2024). Vitamin D supplementation has been found to increase Klotho expression (Haussler et al. 2020), while disturbances in this hormonal axis are common in individuals with increased WWI and metabolic syndrome (Prud'homme et al. 2022). Additionally, the renin‐angiotensin system is involved: angiotensin II suppresses Klotho, and blockade (e.g., with losartan) can upregulate Klotho expression (Janić et al. 2019; Lim et al. 2014; Haussler et al. 2020). Genetically, sex‐specific variants and regulatory loci for WC and waist‐hip ratio highlight the role of genetics in fat distribution and Klotho regulation (Randall et al. 2013; Winkler et al. 2015; Yaghootkar et al. 2016). Such variants may partly explain both WWI and Klotho variation across populations.

Our findings suggest that WWI could serve as a convenient, noninvasive screening tool in the clinical setting for the early identification of individuals at risk of accelerated aging or metabolic dysfunction. Since WWI calculation relies on simple anthropometric data, it is easy to implement in routine health examinations. Combined with serum Klotho measurements in high‐risk populations, this may allow clinicians to initiate preventative strategies earlier, particularly in groups such as older adults or those with chronic diseases.

For clinical application and prevention, personalized intervention strategies focusing on reducing central obesity and maintaining hormonal balance may be especially beneficial for women with high WWI. Health education and screening should be tailored according to sex, and future research should examine estrogen, genetic background, and the molecular pathways linking fat distribution and Klotho regulation (Heid et al. 2010; Randall et al. 2013; Winkler et al. 2015; Yaghootkar et al. 2016).

However, we need to acknowledge that predicting Klotho levels based solely on a single measurement of WWI oversimplifies the complexity of aging and its biomarkers. Additionally, there are other potential confounding factors beyond those mentioned in this study, such as physical activity and diet, that need to be further investigated to clarify the relationship between WWI and serum Klotho levels.

Due to the cross‐sectional nature of this study, we cannot establish causality between WWI and serum Klotho levels. The observed associations require further investigation through longitudinal studies.

## 5 Conclusion

Utilizing a nationally representative sample, this study revealed a linear association and a dose–response relationship between WWI and serum Klotho levels among adults in the United States. The inverse relationships continued to be significant across different subgroups. Additional research is required to confirm these results in different populations and to gain a deeper understanding of the underlying mechanisms.

## Author Contributions

**Jingjing Chen:** conceptualization (lead), data curation (lead), methodology (lead), project administration (lead), writing – original draft (equal), writing – review and editing (equal). **Qingyi Zeng:** investigation (equal), writing – review and editing (supporting). **Li Liu:** data curation (equal), writing – original draft (equal). **Yilan Li:** data curation (equal), writing – original draft (equal). **Anning Wang:** data curation (equal), writing – original draft (equal). **Yingqi Yi:** data curation (equal), writing – original draft (equal). **Zhanglan Wang:** data curation (equal), writing – original draft (equal). **Weihong Sun:** data curation (equal), writing – original draft (equal). **Wei Zhou:** data curation (equal), writing – original draft (equal). **Yun Ye:** data curation (equal), writing – original draft (equal). **Wei Li:** funding acquisition (lead), project administration (lead), writing – review and editing (equal).

## Ethics Statement

The studies involving humans were approved by the GWAS data used in this study were acquired from studies that received approval from relevant ethical review boards. Hence, ethical approval was not necessary for this study. The studies were conducted in accordance with local legislation and institutional requirements.

## Consent

The participants provided their written informed consent to participate in this study.

## Conflicts of Interest

The authors declare no conflicts of interest.

## Data Availability

The original contributions presented in the study are included in the article/Supporting Information (https://www.cdc.gov/nchs/nhanes/index.h). The study was not pre‐registered on any platforms.
